# Is Virtual Reality Cue Exposure a Promising Adjunctive Treatment for Alcohol Use Disorder?

**DOI:** 10.3390/jcm10132972

**Published:** 2021-07-01

**Authors:** Zofia Lebiecka, Tomasz Skoneczny, Ernest Tyburski, Jerzy Samochowiec, Jolanta Kucharska-Mazur

**Affiliations:** 1 Department of Health Psychology, Pomeranian Medical University, 26 Broniewski Str., 71-457 Szczecin, Poland; 2 Department of Psychiatry, Pomeranian Medical University, 26 Broniewski Str., 71-457 Szczecin, Poland; tskoneczny@gmail.com (T.S.); samoj@pum.edu.pl (J.S.); jola_kucharska@tlen.pl (J.K.-M.); 3 Institute of Psychology, SWPS University of Social Sciences and Humanities, 10 Kutrzeby Str., 61-719 Poznan, Poland; etyburski@swps.edu.pl

**Keywords:** virtual reality, mental health, substance use disorders (SUD), alcohol use disorders (AUD), assessment, treatment, intervention

## Abstract

This narrative review presents recent developments in virtual reality (VR)-based interventions for alcohol use disorders (AUDs). The latest advances in mental healthcare hail an imminent cyber revolution, ushering in novel treatment options, with immersive virtual technology at the very forefront of expected change. With an aim to (a) provide a background on VR use in mental healthcare of AUD patients, (b) summarize existing evidence on conventional approaches to the treatment of AUDs and a trending paradigm shift towards VR applications in their management, and (c) describe key issues and future directions in research on craving assessment and VR cue-induced therapy in AUDs, a search for experimental and meta-analytic evidence was performed in six databases: PubMed and EBSCO (Medline, ERIC, PsychINFO, Academic Search Ultimate, and Health Source: Nursing/Academic Edition). Pooled results were screened for eligibility, and relevant papers were selected for inclusion. The analysis revealed VR’s promising effects in the treatment of AUDs. Its remarkable potential to simulate cues underlying subsequent addictive behaviors makes its application in the assessment and treatment of AUDs an attractive alternative to researchers and clinicians alike. Nevertheless, more evidence is needed before virtual reality cue exposure therapy (VR-CET) can become a clinical standard of care.

## 1. Introduction

The latest developments in mental healthcare seem to hail a fast-approaching cyber revolution, giving rise to novel opportunities within existing treatment options, with immersive virtual technology at the very forefront of expected change. As a powerful tool enhancing learning and behavior modification, virtual reality (VR) shows definite promise as an add-on to conventional clinical management of an entire array of mental health problems [1,2,3]. A commonly echoed advantage of immersive virtual technologies over the standard clinical approaches is their ability to far more closely mimic real-life settings, mostly due to their lack of limitations in the delivery of external cues or creation of interactive computer-generated worlds. Relatedly, such a shift away from the customary clinical context and towards the realm of digital-based sensory and cognitive experience offers tighter control over presented stimuli and experimental conditions, endless stimulation opportunities far beyond what any conventional lab setting could ever strive to recreate, consistent treatment protocols, and more extensive availability, not to mention the potential to reach the consumer market and enter the home.

Dating back to the 1980s, applications of basic VR hardware were mostly restricted to laboratory settings [4] and, primarily due to the technological limitations of their times, raised some important questions concerning their true capacity to provide the experience of real immersion and interaction. Nonetheless, the wake of modern generation head-mounted displays and related gear, attributable to the contributions of global IT manufacturers and software vendors, has made such issues largely a thing of the past, enabling new and much more advanced technologies, a breakout of laboratories, and a deep dive into individual use for purposes exceeding mere entertainment. Indeed, VR-related products are now developed by circa 230 companies worldwide [5], with a marked and growing interest from the video game industry in the applications of their products not only in the originally dedicated fields, but also expanding their operations into the education, military, or medical sectors. Therefore, various domains of the health sector have become a novel area of interest for VR tech creators, with psychiatry and mental health disorders unquestionably among them [3,6].

Through high-fidelity simulations of real-time experience-delivered images, sounds, and other sensations imitating a user’s physical presence in a thus augmented or extended reality, VR technology is designed to mimic real-world settings [7]. Incorporating auditory, video, and even haptic feedback, modern systems provide a headset-generated live feed with positional and rotational motion tracking, while dedicated controllers, gloves, or chairs elicit tactile sensations and hence the illusion of actually “being there”, thereby rendering fuller immersion into the 3D virtual world. Therefore, applied in the medical field and no more limited by particular environmental or perceptual setting constraints, VR offers the potential to overcome such limitations of conventional diagnostic and treatment approaches, such as reduced ecological validity or the lack of standardization, control, and generalization tools.

Restrictions linked to staging lifelike settings, however, clearly reduce the scope of interventions offered to patients in standard clinical conditions. Stripped of such limitations, VR shows promise in creating true-to-life simulations of such difficult situations, in which patients can be monitored and navigated to develop more appropriate and healthy responses in an individual, tailor-made, non-threatening, and repeated fashion, depending on the particular type of deficits they are manifesting. The idea behind such a lifelike approach, albeit extending beyond what is feasible in a traditional lab setting, is that the less threatening, easier to manipulate, repeated, and controlled VR conditions enable learning that will be easily transferable into the real-world experience of patients. For even though the reality is “fake”, the elicited mental and physical responses, much like all acquired skills and psychological resources, are not. Clearly, however, if the exposure conditions can be well-controlled and easily manipulated, full VR potential will be apparent not only in improving treatment strategies, but also assessment, diagnosis, and evidence-based capturing of the underpinnings of mental health disorders [8].

Indeed, the past few decades have brought evidence of VR efficacy in the management of a wide range of psychiatric conditions, proving a successful solution for anxiety disorders, depression, psychosis, and eating and substance use disorders [1,5], with markedly fewer reports of its applications in the treatment of or research into other mental health problems, like sexual [9] or sleep disorders [10]. Interestingly, VR-based treatment options compare quite well with corresponding person-to-person therapies, with evidence of efficacy matching that of the existing approaches [11], generalization to the real world [12], and durability of the effects [13].

Exposure therapies involving VR technology are also quite extensively used in the treatment of problematic behaviors, i.e., drug misuse, alcohol dependence, or pathological gambling. The majority of the available evidence demonstrates the virtual environment’s great capacity to induce cravings, amongst others, for alcohol [14], cocaine [15], or nicotine [16,17,18]. Therefore, there are reports of VR applications in the treatment of a range of addictive disorders, including nicotine and alcohol dependence, gambling, or Internet gaming disorders [7,19], with interventions based on cue exposure for smoking [20,21,22] and methamphetamine addiction [23,24,25], or approach and avoidance training for alcohol dependence [6,26]. Nevertheless, studies to date concerning the most widely researched nicotine dependence seem to yield somewhat conflicting results, suggesting a comparable effect of virtual reality cue exposure therapy (VR-CET) to conventional cognitive-behavioral therapy (CBT) interventions [27], greater efficaciousness of CBT-adjuvant VR-CET [20], or no increase in efficacy in response to add-on VR-CET protocol combined with an elevated relapse risk elsewhere [22]. Such findings therefore dictate a rather cautious approach to utilizing VR-CET protocol, and augmenting instead of replacing conventional therapies still seems to be a more encouraged paradigm [7].

Given VR’s remarkable potential to simulate cues that trigger cravings responsible for subsequent addictive behaviors, its application in the management of substance use disorders seems to attract growing attention of both researchers and clinicians alike. In this review, we summarize the current knowledge on craving assessment and cue-induced therapy in the mental health sector, with a particular focus on alcohol use disorders (AUDs), using VR technologies. To this end, pooled literature search results screened for eligibility and relevant for inclusion in this review were analyzed to meet the following specific aims: (a) to provide a background on VR use in mental healthcare for adults with AUD, (b) to summarize the existing body of evidence on conventional approaches to the treatment of alcohol use disorders and a trending paradigm shift towards VR applications in the management thereof, and (c) to describe key issues and future directions in research on craving assessment and cue-induced therapy in AUDs using virtual, augmented, or extended reality technologies.

## 2. Methods

A literature search was conducted based on Ferrari’s [28] suggestion for narrative reviews and a basic methodology used by Freeman et al. [1]. To this end, a search of PubMed and five databases from EBSCO (Medline, ERIC, PsychINFO, Academic Search Ultimate, and Health Source: Nursing/Academic Edition) for both experimental and systematic review evidence published up to June 2021 was performed. The inclusion criteria were: a focus on alcohol-related disorders; empirical or systematic reviews (including meta-analytical studies and reviews); considered a form of immersive VR; and published in a peer-reviewed journal in the English language. We did not consider personality disorders, childhood-onset disorders, or the effects of VR applications that were not designed as interventions. We used the following search terms: ((VR or virtual reality) and (substance use disorder* or alcohol use disorder* or alcohol) and (cue exposure treatment or therapy)).

Two researchers (Z.L. and E.T.) independently screened records to be included. Any discrepancies were resolved by consensus. Search results were screened for eligibility first by title and abstract, then by full-text review. Reasons for exclusion were recorded for each excluded study. In addition, we performed a manual search of the references of articles selected for inclusion in this paper.

## 3. Results

A total of 548 studies were identified from the literature searches (310 in EBSCO and 238 in PubMed). After excluding duplicates, 241 studies were identified and 50 papers were selected for further screening. Finally, 29 articles (13 experimental studies and 16 reviews) were included and analyzed in this narrative review.

### 3.1. Virtual Reality and Alcohol Use Disorders

As a major public health issue of increasing global importance, substance use disorders (SUDs) generate substantial health consequences alongside significant social and economic burden to both individuals and societies worldwide. SUDs remain difficult to treat, their relapse rates reaching an average of 50% despite the use of available treatment [29]. A particularly significant source of harm is represented by addiction to alcohol, a causal factor in > 200 disease and injury conditions, the harmful use of which contributes to 3 million deaths every year, i.e., 5.3 % of all deaths worldwide, with even more staggering numbers reaching 13.5% of the total mortality in the age group 20–39 years [30].

Even though the major addictive compound in AUDs is very well-known and has been studied from both pharmacological and toxicological perspectives, relapse prevention and treatment thereof remain a challenge, as most psycho- and pharmacological therapy approaches continuously fail to warrant satisfactory success rates. While addiction medicine relies quite heavily on the combined use of medication and what can be collectively labeled as cognitive–behavioral interventions, increasing attention seems to be dedicated to establishing novel clinical paradigms with a potential to improve such combinations, thus expanding on both assessment and treatment options (c.f., [7,31]). Already well-recognized in neurosciences and psychology, modern computer technologies, in particular VR, may prove to be valuable supplementary tools in the assessment and treatment of AUDs.

The ICD-10 guidelines for alcohol dependence include (a) a strong desire or sense of compulsion to take the substance, (b) difficulties in controlling substance-taking behavior, (c) physiological withdrawal, (d) evidence of tolerance, (e) progressive neglect of alternative pleasures or interests, and (f) persisting with substance use despite clear evidence of overtly harmful consequences [32]. This strong desire or sense of compulsion to take the substance, i.e., craving, is not only a significant diagnostic criterion, but also a factor considered predictive of relapse [33,34], and therefore is a phenomenon worthy of further investigation in the context of assessment and treatment models.

Due to the fact that the works on the eleventh revision of the ICD have been finalized and the ICD-11 has been released for use, it seems worthwhile to at least make a mention of how it affects AUDs. Similarly to ICD-10, the eleventh revision proposes alcohol dependence disorder as the central diagnostic entity concerning pathological alcohol use. The ICD-11 considers it a disorder of regulation of alcohol use stemming from its repeated or continuous consumption, the key feature being a strong internal drive to drink understood in terms of impaired control, frequently but not necessarily combined with a feeling of urge or craving [35]. Time will tell if the changes in the understanding of AUDs heralded in ICD-11 relative to ICD-10, though only minute, will therefore enforce different and potentially more efficacious therapeutic approaches in the future. Nevertheless, the sensation of craving seems to remain central to the concept of pathological alcohol use.

A neurocognitive perspective deems it fit and proper to understand addictions in terms of a certain imbalance between impulsive (or reflexive) and reflective decision-making [36]. Behind the capacity to resist craving and make adaptive decisions stands the reflective system, dependent on cognitive inhibitory control and delayed gratification. In turn, responsible for the reflexive processes, i.e., those linked to impulsivity and risky behaviors, are all of the automatic motivational and emotional responses to reward, yielding faster approach action tendencies [37]. Additionally, as a multi-faceted construct, impulsivity may be further differentiated into impulsive choice (of a smaller, yet more immediate reward over a delayed one) and impulsive action (stemming from the inability to stop a dominant behavior [38]). Both impulsive choice and action may be due to reward-seeking or poor (even lacking) inhibitory control. Compulsive substance seeking combined with a neglect of controlled decision-making in patients with AUDs are explained by means of deficits and perturbations within both reflective and reflexive systems.

A socio-environmental approach, on the other hand, implicates a number of social and environmental cues and certain individual sensitivity to them as involved not only in the very development of AUDs, but also processes of recovery and maintenance of abstinence. It therefore seems that particular substance-related settings (pubs, bars, nightclubs) and social interactions may first trigger craving and ultimately relapse in even abstinent individuals [39]. Remarkably, this vulnerability to alcohol-related cues alongside the anticipation of reward are thought to potentially translate into a neural response in the form of reward pathway activation. There is neurobiological evidence that SUDs (c.f., [40,41,42,43,44,45]), and so by extension also AUDs, reflect pathology of neuroplasticity and that substance-related socio-environmental cues provoke behavioral and underlying neurobiological responses.

### 3.2. Effectiveness of Cue Exposure Therapy

Given the essential role of (largely cue-induced) craving in the mechanisms of AUDs, its assessment becomes crucial for the identification, management, treatment design, and effects. Various approaches cited in the literature have been applied to investigate alcohol cravings, focusing on their neurobiological and psychophysiological underpinnings [46,47] with the use of paper and pencil tools [48], cognitive and/or behavioral tasks [49,50], or, finally, the cue exposure paradigm [51].

Cue exposure is generally performed in vivo through relevant imagery or via virtually generated settings. The majority of available evidence-based methods of inducing craving include substance-associated visual cues, such as images or videos, alone or combined with other sensory stimuli (usually auditory or tactile). To date, most conventional cue exposure therapy (CET) approaches for AUDs have seemingly been based on visual delivery of alcohol-related stimuli, showing limited success in terms of reduced alcohol consumption rates [52]. However, conventional CET designs seem to yield somewhat mixed results in terms of their efficacy when used in AUD clinical populations [53,54]. Restricted efficacy of traditional methodologies has been linked to the clinical settings, which seemed to preclude the emergence of approach biases, thus keeping the impulsive system inactive [55,56]. One explanation may be that, although visual cues remain relevant to substance-seeking behavior and could therefore successfully induce craving, when delivered in artificial, alcohol consumption-unrelated settings, they may not necessarily provoke responses otherwise linked to either social or environmental cues (specific settings or social interactions).

Evidence now affirms that intervention in both drug abuse vulnerability and reactivity to environmental or contextual cues is useful in support of pharmacotherapy in the management of SUDs [57]. As described above, interventions based on CET, delivered either conventionally or via augmented reality technologies, have been used extensively in patients with social and specific phobias, post-traumatic stress disorder, and generalized anxiety disorders [58]. However, currently available results suggest a rather mitigated success of CET in treating SUDs. Although CETs do demonstrate interesting therapeutic potential, they do not seem to yield treatment outcomes considered significantly superior to other CBT-based approaches [22], not to mention existing methodological issues in CET research that need to be addressed before any considerable benefits can be announced [59]. Novel psychological treatments involving e.g., VR paradigms, may represent an answer to this issue, as they are designed to build on the technology’s capacity to provide the experience of social immersion and interaction within this given context [60,61].

### 3.3. Effectiveness of Exposure to Virtual Reality Environments

Notably, there seem to be differences in how craving is elicited through the sole exposure to VR environments and how it is modified by possible interactions with avatars across diverse VR paradigms. The former experimental settings may involve images of people performing substance-related actions and VR scenarios of bars, clubs, or parties, with the subjects assuming the roles of mere observers. In turn, more specialized paradigms engage the subjects in interactions with or even control over (elements of) the virtual environment. These experimental designs, however, have been considered somewhat lacking in terms of standardized forms of delivery and subjects’ performance in response to the presented VR setting (c.f., [62,63]). In AUDs, one of the critical areas for exploration is the differentiation between social and pathological drinkers [64]. The fact that VR environments are successful in inducing cravings for alcohol was demonstrated by Ryan, Kreiner, Chapman, and Stark-Wroblewski [65], who investigated the use of VR cues in binge vs. non-binge drinkers. Compared to their non-binge counterparts, binge drinkers reported significantly higher alcohol cravings in two out of the four proposed VR protocols (party and kitchen scenarios). Remarkably, another one of the applied settings—the bar scene—proved to elicit increased cravings within both groups. Bordnick et al. [66] investigated the effect of the VR paradigm in AUD subjects, exposing them to neutral and alcohol-related VR environments. Alcohol-related cues combined with interactions with a virtual bartender and ordering their drink of choice proved to induce cravings much more successfully relative to neutral scenarios. Other studies included elements of social pressure, where avatars urged subjects to engage in alcohol use behavior. While the effect of such scenarios on subjective craving levels has indeed been reported [14,67], findings concerning the exact mechanism underlying craving (and its triggers) remain inconsistent, and data are relatively scarce. Particular difficulty lies in differentiating between social craving from context craving [7].

VR cue exposure proves efficacious in craving induction, through cues typically associated with alcohol consumption (including different types thereof, i.e., proximal: alcohol bottles, contextual: bar, or complex cues: party scenario [14,65,66,68]). Therefore, a VR scenario of a first-person recollection of attending a party resulted in reduced alcohol cravings [69]. Virtual technology may also contribute to the prognostication of future beverage choices (alcoholic vs. non-alcoholic) [70]. There are also reports that VR-CET-based training may lead to changing approach bias via visual and auditory stimuli, thus reducing automatic action tendencies toward alcohol [26]. Elsewhere, a similar task was reported to be a useful and accurate measure of craving in social drinkers, mostly due to its ability to provide realistic scenarios and the sense of immersion in virtual environments [71]. In their systematic review on the use of VR in the assessment and treatment of addictive disorders, Segawa et al. [7] reported findings from three controlled Korean studies evaluating virtual exposure therapy (VET [72,73,74]). Compared to conventional CBT, VET delivered in the form of 10 biweekly sessions proved more effective in reducing alcohol cravings in 38 investigated male participants. Each therapist-guided session consisted of relaxation, craving, and aversive exposure [73]. The same protocol, however, revealed no links between reduced craving and brain metabolism in FDG-PET [74]. Elsewhere, in the first phase of a clinical trial comparing VR-CET and TAU involving CBT-based interventions, Ghiţă et al. [75] demonstrated equivalent effects of both approaches in reducing craving and anxiety, suggesting the potential of VR technology in the treatment of SUDs. Due to a small sample size, their results should, however, be construed as preliminary at most. Quite remarkably, there is evidence that even a single VET session can effectively contribute to craving reduction in heavy social drinkers compared to light alcohol consumers [72]. Furthermore, Hernández-Serrano et al. [31] reported on improved outcomes in patients whose treatment was complemented with VET relative to the treatment as usual (TAU) group, thus suggesting benefits of add-on VR-CET intervention in the management of AUDs.

Interestingly, there is evidence of beneficial effects of a partially immersive game-based VR intervention for recovery from alcohol use, countering alcohol cues through movements [76]. Found engaging and fun by its users, the game was reported to support recovery efforts, reducing alcohol consumption during the intervention period and suggesting its utility as a potentially effective adjunct to TAU. A similar approach was examined via a feasibility study aimed to develop a cue exposure-based gaming tool enhanced with virtual reality for the treatment of AUD to facilitate extinction and decision-making training [77]. The tool’s very good reception, high usability ratings (irrespective of participants’ computer experience), low cost, availability, and compelling nature were considered by the authors as good predictors of its utility as an add-on to TAU in the therapy of AUDs.

An interesting conclusion emerged from a systematic review by Durl et al. [78], who found VR to be underutilized in alcohol studies despite the promise it seems to show across its various applications, including CET. Nevertheless, the authors acknowledge the limited relevance of the findings due to the relative scarcity of evidence of the longitudinal effects and efficacy of VR-assisted therapies, thus encouraging further long-term empirical research. Therefore, no matter how encouraging this preliminary data on VR use is in the context of AUD management and how much evidence emerges that VR-CET is effective in treating addictions [79], VET’s efficacy and evidence in terms of alcohol dependence reduction still require further investigation.

## 4. Risks, Challenges, and Future Directions

Not to fall prey to excessive and uncritical enthusiasm linked to VR use in therapeutic settings and to avoid publication bias, it is advisable to mind potential risks of its application. Following Parsons [80], those may include (a) cybersickness, (b) superrealism, experience intensification, and information overload, (c) depersonalization, derealization, and dysfunctional re-entry into the real world, (d) unintended outcomes in vulnerable populations, and (e) misconceptions concerning the true benefits of VR exposure (or lack thereof) in vulnerable cohorts. In addition, the issues of limited clinician training in terms of privacy, electronic security, or legal implications, consistent upgrades of available technologies necessitating user training (which may particularly affect vulnerable populations), or the costly nature of hard- and software, especially of the highly immersive methods (e.g., head-mounted displays), are raised. For the beneficence of patients, it is therefore essential that clinicians balance the benefits of immersive simulations offered by novel technological solutions and the true costs of applying VR to the interventions.

Furthermore, despite the growing body of evidence regarding VR use in mental health, there still remain certain issues to be addressed, as the development of novel technologies has led to the emergence of new queries and challenges. Amongst those are questions of limited methodological quality, piecemeal evidence, potentially constrained reporting of negative outcomes [1], or general gaps in reporting [29]. The questions concerning learning transfer from VR to the real world, VR’s utility for complex psychological treatment techniques (as opposed to the simple ones like CET), and its real-life benefits for patients, especially surmounting those of treatment as usual, are still timely and valid. Although there is no doubt that both the science and technology behind VR are evolving, leading to more affordability, its cost is likely still a barrier not only for patients, but also the majority of healthcare providers. On top of that, most applications designed for use in treatment were developed to aid in research, and therefore are not available for the general public [81]. Future research should therefore focus on better control for human and avatar interaction, improved and cost-effective technology for enhanced user experience (i.e., specialist programming expertise), extended scenarios and social interactions, more rigorous methodological approaches meeting the standards expected in clinical research (e.g., comparability of research designs and results), or issues such as self- vs. therapist-administered therapies.

Two other interesting research directions seem to be the so-called co-creation [82], i.e., a collaborative involvement in the final intervention design of end-users and broader stakeholder groups, and augmented reality (AR), a rapidly emerging technology that has the potential to take VR studies to the next level and pave the way to future research on this novel and efficacious treatment option for mental health disorders. Superimposing virtual objects onto real-world backgrounds via diverse types of displays, including smartphones, tablets, or headsets in real-time, AR demonstrates a potential especially to be used for extinction-based therapies (administered also in substance use disorders), taking them beyond the clinic [83]. VR and AR alike may therefore hold promise to deliver what is beyond any real-life clinical setting, but the question remains whether we can find a way to make the right use of it.

## 5. Limitations

In this paper, we have shed some light on the current body of evidence on VR applications in the mental health sector, with a particular focus on AUDs. The work is not, however, free from limitations. First and foremost, it is a narrative and not a systematic review, which may be criticized for a less rigorous methodological approach. The rationale behind choosing this form of expression was to offer a broader and up-to-date outline and overview of available meta-analytic and original research results concerning the rapidly evolving knowledge about VR applications in mental healthcare settings. Even though it is stripped of the rigor of a systematic review, we have made every effort for this paper to present an objective and accurate synthesis of the relevant literature in a comprehensive manner, all the while remaining unbiased in our critique thereof.

## 6. Conclusions

VR immersion techniques seem to offer a good alternative to conventional cue-provoked treatment approaches, and have proven to successfully integrate simulated social interactions in such paradigms. Although both experimental and meta-analytic evidence supports the effect of exposure to VR cues as a promising add-on to standard CET approaches in various mental disorders, and VET in AUD is reported to elicit cravings by exposure to and interaction with environments related to typical alcohol consumption scenarios, its beneficial effect and superiority in the assessment and management of AUDs needs to be further validated, as high-fidelity simulations have been suggested to offer the potential for both desirable and unwanted outcomes. That said, VR-based approaches definitely bear promise in developing applications promoting individualized and accessible care in not so distant future of psychiatry and psychology.

## Data Availability

Materials of the review reported here are available from the corresponding author on reasonable request.
